# Dopamine D2 receptor gene polymorphisms and externalizing behaviors in children and adolescents

**DOI:** 10.1186/s12881-018-0586-9

**Published:** 2018-05-02

**Authors:** Osmar Henrique Della Torre, Lúcia Arisaka Paes, Taciane Barbosa Henriques, Maricilda Palandi de Mello, Eloisa Helena Rubello Valler Celeri, Paulo Dalgalarrondo, Gil Guerra-Júnior, Amilton dos Santos-Júnior

**Affiliations:** 1 Department of Psychiatry - Faculty of Medical Sciences (FCM), State University of Campinas (Unicamp), Campinas, SP Brazil; 2 Laboratory of Human Genetics - Center for Molecular Biology and Genetic Engineering (CBMEG), Unicamp, Campinas, SP Brazil; 3 Growth and Development Laboratory - Center for Investigation in Pediatrics (CIPED), FCM – Unicamp, Campinas, SP Brazil; 4 Department of Pediatrics - Pediatric Endocrinology Unit, FCM – Unicamp, Campinas, SP Brazil; 5 Rua Tessália Vieira de Camargo, 126, Cidade Universitária Zeferino Vaz - Campinas, São Paulo, ZIP Code: 13083-887 Brazil

**Keywords:** Genetic polymorphism, Dopamine, Behavior, c.957C > T polymorphism, c.-585A > G polymorphism

## Abstract

**Background:**

Dopamine is involved in several cerebral physiological processes, and single nucleotide polymorphisms (SNP) in the dopamine D2 receptor gene (*DRD2*) have been associated with numerous neurological and mental disorders, including those involving alterations in cognitive and emotional processes.

**Methods:**

The aim of this study was to evaluate the association between the SNPs c.957C > T (rs6277) and c.-585A > G (rs1799978) in the *DRD2* gene and behavioral characteristics of children and adolescents based on an inventory of the Child Behavior Checklist (CBCL). Children and adolescents between 8 and 20 years old who were clinically followed-up were genotyped for the SNPs c.957C > T and c.-585A > G, and related to data of the CBCL/6–18 scale assessment performed with the help of caregivers. The chi-squared test was used to assess the differences in the frequencies of the C and T alleles in the polymorphism c.957C > T and of the A and G alleles in the polymorphism c.-585A > G with respect to the grouped CBCL scores at a significance level of 5%. Multiple logistic regression models were performed, to control whether sex and/or ethnicity could influence the results.

**Results:**

Eighty-five patients were assessed overall, and the presence of the T allele (C/T and T/T) of *DRD2* c.957C > T polymorphism was found to be significantly associated with the occurrence of defiant and oppositional problems and with attention and hyperactivity problems. There were no associations detected with polymorphism *DRD2* c.-585A > G polymorphism. Both SNPs were in Hardy-Weinberg-equilibrium.

**Conclusions:**

Although the findings of this study are preliminary, due to its small number of participants, the presence of T allele (C/T, T/T) in c.957C > T SNP was associated with difficulty in impulse control, self-control of emotions, and conduct adjustment, which can contribute to improving the identification of mental and behavioral phenotypes associated with gene expression.

## Background

The dopamine D2 receptor gene (*DRD2*), located at chromosome 11q23.1, is involved in several cerebral physiological processes, including behavior inhibition and externalizing conditions, i.e., aggression and symptoms such as oppositional defiance, conduct problems, and attention deficit/hyperactivity [1–7].

The single nucleotide polymorphism (SNP) c.957C > T (rs6277) is located on the seventh exon of *DRD2* gene at the 319 codon that codes a proline [8]. Individuals with C/C homozygous genotypes, associated with a higher density of extrastriatal D2 receptors, were shown to be more efficient in inhibiting unwanted action tendencies and showed increased reward responsiveness after stress induction compared to T/− carriers [5, 9]. The presence of the T allele is associated with a reduction of the translation and stability of the messenger RNA (mRNA), and with a reduction in protein synthesis by up to 50% in comparison with activity of the C allele [10–12].

The functional consequence of SNP c.-585A > G (rs1799978) is currently unknown; however, it is suspected to regulate the expression of the *DRD2* gene due to its location on the promoter region [13]. The G allele has been associated with conditions related to impulsiveness [14].

In this study, we tested the hypothesis that the T allele of the c.957C > T (rs6277) polymorphism and/or the G allele of the c.-585A > G (rs1799978) polymorphism are associated with the occurrence of externalizing behaviors among children and adolescents under psychiatric treatment.

## Methods

This was a descriptive cross-sectional study, with a sample composed of patients attending the Psychiatric Outpatient Clinic of Children and Adolescents, Department of Medical Psychology and Psychiatry, Clinical Hospital and School of Medical Sciences, University of Campinas (Unicamp) from March 2014 to August 2015. The center provides a service that typically cares for severe cases, most of whom are under a regime of psychotropic drugs, requiring a more complex level of mental health attention than those receiving primary health care.

In addition to information about the use of medications and diagnoses registered on medical records, the inventory of the Child Behavior Checklist for the age range of 6–18 years old (CBCL/6–18) was used for qualification of the presence and severity of the mental and behavioral symptoms [15]. The CBCL/6–18 is a standardized questionnaire designed by Achenbach in 1991, answered by the parents or caregivers, which is used to assess the behaviors of children or adolescents [16] and was validated for application in the Brazilian population [17]. The CBCL/6–18 is widely recommended as one of the most effective tools for the qualification of parental responses related to childhood behavior [18]. Such an inventory provides a profile of behavioral and emotional problems in syndromic groups, but has not been validated as a diagnostic instrument [18, 19]. As the CBCL/6–18 does not evaluate neurocognitive problems, individuals with severe or profound intellectual disability were not included in this study, because their behavioral symptoms would not be differentiable between those associated with intellectual disability itself and those due to other disruptive behavioral disorders. The emotional/behavior problem section of the CBCL/6–18 has 118 items [20]. The adult respondent has to attribute the following scores to each problem: 0, not true; 1, somewhat true or rare; and 2, very true or very frequent over the last six months [17]. Analysis of the CBCL/6–18 scores was conducted with the software of the Achenbach System of Empirically Based Assessment [17], and the results were grouped into three kinds of profiles: syndrome-based scores, which evaluate symptoms for each of the main clusters of mental health problems; scales that mainly group psychological symptoms according to internalizing and externalizing dimensions; and scales consistent with DSM-IV scales [19]. The following DSM-oriented scales were used: “anxiety problems”, “somatic problems”, “attention deficit hyperactivity disorder problems”, “opposition and challenging problems”, and “conduct problems” [19].

The results obtained from the CBCL/6–18 responses from parents or other adult caregivers, and not the children/adolescents, were used to classify the children/adolescents into the following categories: “normal” (behavior scores < 67, total scores < 60), “borderline clinical range” (behavior scores 67–70, total scores 60–63), and “clinical range” (behavior scores > 70, total scores > 63) [19].

The genomic DNA samples were obtained from 8 mL of total peripheral blood collected in tubes with EDTA (0.6 M), pH 8.0 as anticoagulant. Genomic DNA was purified from peripheral leukocytes according to standard protocols by lysing with proteinase K, extracting with phenol/chloroform, and precipitating with ethanol [21]. To determine genotypes for c.957C > T (rs6277) and c.-585A > G (rs1799978) in the *DRD2* gene, real-time PCR using *TaqMan®* allelic discrimination assay (Applied Biosystems, Foster City, CA - USA) and primers available from SNP Genotyping Assay (Applied Biosystems, Foster City, CA - USA) were used. The total volume for all reactions was 7 μl containing *TaqMan Genotyping PCR Master Mix* 2X (3.5 μl) and SNP *Genotyping Assay* 40X (0.175 μl), MiliQ water (2.325 μl) and 10 ng of each genomic DNA (1 μl). Reactions were performed in 96-well optical plates (0.1 ml MicroAmp, Applied Biosystems) and submitted to the following temperatures and cycles: a first cycle of 10 min denaturation at 95 °C followed by 40 cycles of 15 s at 95 °C and 1 min extension at 60 °C. Amplification reactions were performed in a 7500 *Fast Real-Time PCR System (*Applied Biosystems). Validation studies demonstrate that the Applied Biosystems 7500 System SDS is a robust, reliable, and reproducible system for performing DNA quantification [22]. Data were recorded and analyzed using 7500 System Sequence Detection Software*©* (SDS).Descriptive and chi-square statistical analyses were carried out using SPSS version 22 (IMB Co., Armonk, NY, USA). For relevant associations, multiple logistic regression analyses were carried out using Stata/SE version 14.1 (StataCorp LP, College Station, TX, USA). They were performed to control whether the associations were due to sex or ethnicity differences. Each model of multiple logistic regression analysis used, as dependent variable, the emotional/behavioral problem evaluated by CBCL 6/18 which had significant statistical association with the presence/absence of the T allele of the rs6277 SNP of the *DRD2* gene. Sex, ethnicity and the presence/absence of the T allele itself were the independent variables.

Hardy-Weinberg equilibrium was evaluated by the Haploview software (BROAD Institute) [23, 24] to determine if the allele frequencies of the SNPs assessed in the study population were balanced and therefore applicable for association studies. For the statistical analysis, the CBCL/6–18 results were grouped into two distinct groups of two categories each: in the first group, the results were compared between those without alterations (“normal”) versus those with alterations (“borderline clinical range” plus “clinical range”); in the second group, the results were compared between those without alterations or with few alterations (“normal” plus “borderline clinical range”) versus those with substantial alterations (“clinical range”). The chi-squared test was applied to evaluate the differences in the C and T allele frequencies for the polymorphism c.957C > T and in the A and G allele frequencies for the polymorphism c.-585A > G in relation to the grouped scores of the CBCL/6–18. The significance level adopted was 5%.

## Results

This study comprised 85 patients with a mean age of 13.4 ± 2.7 years. Sixty-five patients (76.5%) were male. The sample was composed of 61 (71.8%) Caucasian individuals, 15 (17.6%) bi-racial (African and Caucasian individuals), 8 (9.4%) African individuals, and 1 (1.2%) Asian individual. The majority of the CBCL/6–18 assessment respondents were women (84.7%), 54 (75%) of which were the biological mothers. The other respondents included fathers, grandparents, and shelter caregivers that accompanied the child or adolescent. Three participants had already been hospitalized in a psychiatric inpatient ward, due to severe disruptive behavior (one with 11 days of hospitalization, one with 14 days and the third with 17 days), but all of them had already been discharged by the time of the beginning of the study.

All patients were receiving risperidone therapy, and 19 patients (22.7%) were on risperidone monotherapy. By the time of data collection, all participants were in psychiatric outpatient treatment for externalized psychopathological conditions in which risperidone may be utilized. Antidepressants were also involved in the treatment regimen of 46 (54.1%) cases, followed by psychostimulants in 23 patients (27.1%) and clonidine in 11 patients (12.9%). Anticonvulsants, lithium, benzodiazepines, and other associated antipsychotics accounted for 17.6% (15 patients) of the total use of medicines. The medication had been given for a mean of 34.6 ± 23.5 months.

According to the clinical evaluation made by experienced psychiatrists and collected from the patients’ medical records, the psychiatric syndromes were described as: disruptive/aggressive (42 patients, 49.4%), hyperkinetic (35 patients, 41.2%), depressive (29, 34.1%), intellectual disability (24, 28.2%), autism (20, 23.5%), phobic-anxious (17, 20%), learning disturbances (13, 15.3%), and psychotic (6, 7.1%).

The *DRD2* polymorphisms were in Hardy-Weinberg equilibrium [c.957C > T (rs6277) *p* = 0.4169, c.-585A > G (rs1799978) *p* = 0.246]. Table 1 shows the genotypic distribution, sample allele frequencies, and minor allele frequencies (MAFs) of the SNPs for the study sample and the global population according to the 1000 Genomes Project and HapMap databases. The MAFs of the study population were highly similar to those of the global population for both polymorphic alleles.

**Table 1:** Genotypic distribution and allele frequencies of *DRD2* gene polymorphisms

SNP	Genotype	Allele frequency	MAF^a^ of the study population	MAF^a^ from the global database
rs6277	C/C 43 (50.6%) C/T 37 (43.5%) T/T 5 (5.9%)	C = 123 (72.35%) *T* = 47 (27.65%)	*T* = 27.65%	*T* = 24.4%
rs1799978	A/A 66 (77.6%) A/G 19 (22.4%)	A = 151 (88.82%) G = 19 (11.18%)	G = 11.18%	G = 11.9%

^a^*MAF* minor allele frequency

**Fig. 1:**
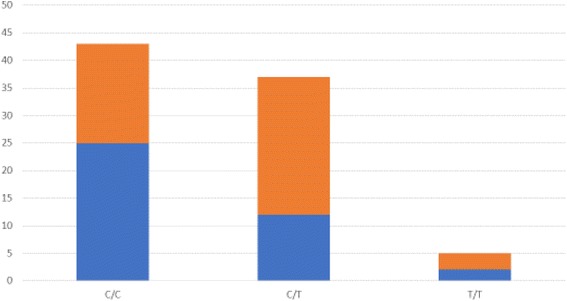
Genotypic distribution of the c.957C>T (rs6277) SNP, regarding challenging and oppositional problems. Above: with alteration; Below: no alteration

**Fig. 2:**
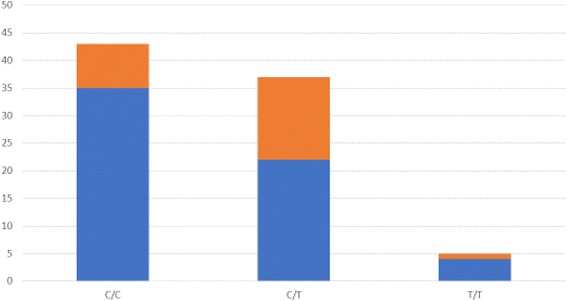
Genotypic distribution of the c.957C>T (rs6277) SNP, regarding attention problems and hyperactivity. Above: substantial; Below: few

Table 2 shows the genotypic distribution of the evaluated SNPs of the *DRD2* gene, regarding sex, age, psychiatric diagnoses and use of psychopharmacological medications. No statistically significant associations were found (*p* > 0.05). Regarding the CBCL/6–18 results, the only significant associations between them and the polymorphisms were between the c.957C > T (rs6277) polymorphism and the occurrence of oppositional defiant disorders, attention problems, and hyperactivity, which are summarized in detail in Table 3. Figures 1 and 2 show the genotypic distribution of the rs6277 SNP (C/C, C/T and T/T) regarding the CBCL/6–18 significant results. There were no significant associations for the SNP c.-585A > G (rs1799978).

**Table 2:** Genotypic distribution of the evaluated SNPs of the *DRD2* gene, regarding demographic data, psychiatric diagnoses and use of psychopharmacological medications

	rs6277			rs1799978^a^	
	C/C	C/T	T/T	A/A	A/G
Sex					
Female	13 (65%)	6 (30%)	1 (5%)	15 (75%)	5 (25%)
Male	30 (46.2%)	31 (47.7%)	4 (6.2%)	51 (78.5%)	14 (21.5%)
Age					
8–10 years-old	6 (37.5%)	8 (50%)	2 (12.5%)	12 (75%)	4 (25%)
11–15 years-old	24 (48%)	23 (46%)	3 (6%)	39 (78%)	11 (22%)
More than 15 years-old	13 (68.4%)	6 (31.6%)	0	15 (78.9%)	4 (21.1%)
Intelligence quotient (IQ)					
Normal	33 (52.4%)	27 (42.9%)	3 (4.8%)	49 (77.8%)	14 (22.2%)
Mild-Moderate intellectual disability	10 (45.5%)	10 (45.5%)	2 (9.1%)	17 (77.3%)	5 (22.7%)
Psychiatric condition					
Disruptive/aggressive	20 (47.6%)	19 (45.2%)	3 (7.1%)	34 (81%)	8 (19%)
Hyperkinetic	16 (45.7%)	16 (45.7%)	3 (8.6%)	24 (68.6%)	11 (31.4%)
Depressive	15 (51.7%)	12 (41.4%)	2 (6.9%)	24 (82.8%)	5 (17.2%)
Autism	14 (70%)	5 (25%)	1 (5%)	16 (80%)	4 (20%)
Phobic-anxious	10 (58.8%)	6 (35.3%)	1 (5.9%)	13 (76.5%)	4 (23.5%)
Learning disturbances	5 (38.5%)	7 (53.8%)	1 (7.7%)	7 (53.8%)	6 (46.2%)
Psychotic	2 (33.3%)	4 (66.7%)	0	5 (83.3%)	1 (16.7%)
Psychiatric medication					
Antipsychotics	43 (50.6%)	37 (43.5%)	5 (5.9%)	66 (77.6%)	19 (22.4%)
Antidepressants	26 (56.5%)	17 (37%)	3 (6.5%)	38 (82.6%)	8 (17.4%)
Psychostimulants	8 (34.8%)	12 (52.2%)	3 (13%)	18 (78.3%)	5 (21.7%)
Clonidine	5 (45.5%)	6 (54.5%)	0	10 (90.9%)	1 (9.1%)
Others	6 (40%)	7 (46.7%)	2 (13.3%)	11 (73.3%)	4 (26.7%)

^a^There were no individuals with the G/G genotype of the rs1799978 SNP of the *DRD2* gene

**Table 3:** Significant associations between the results of the CBCL/6-18 and the rs6277 polymorphism of the *DRD2* gene

	Presence of the T allele (C/T e T/T)^*^	Absence of the T allele (C/C)	*p*-value	χ^2^
Challenging and oppositional problems according to the DSM-IV				
No alteration (0)	14 (35.9%)	25 (64.1%)	0.022	5.265
With alteration (1 and 2)	28 (60.9%)	18 (39.1%)		
Attention problems and hyperactivity by the DSM-IV				
Few alterations (0 and 1)	26 (42.6%)	35 (57.4%)	0.046	3.983
Substantial alteration (2)	16 (66,7%)	8 (33,3%)		

^*^There were no associations between the rs1799978 polymorphism of *DRD2* and any of the CBCL/6-18 results

For challenging and oppositional problems according to DSM-IV, multiple logistic regression models were performed, to test whether the association of the presence of the T allele of the rs6277 SNP of the *DRD2* gene with both oppositional problems and attention problems/hyperactivity could be due to sex or ethnicity differences between groups (Table 4). The presence or absence of the T allele, regarding sex and ethnicity, is shown in Table 4. Sex and ethnicity had no statistical associations with any of the groups of symptoms studied (Table 4) and, after the multiple logistic regressions, the presence of the T allele remained associated both with challenging and oppositional problems (odds ratio = 2.78 [CI95% = 1.15–6.71]; *p* = 0.023) and, with less statistical significance, with attention problems and hyperactivity (odds ratio = 2.69 [CI95% = 1.001–7.24]; *p* = 0.050) (Table 4).

**Table 4:** Presence or absence of the T allele of the rs6277 polymorphism of the *DRD2* gene, according to sex and ethnicity^a^, and logistic regression analyses

	Presence of the T allele (C/T and T/T)	Absence of the T allele (C/C)		*p*-value	χ^2^	
Sex						
Female	7 (35%)	13 (65%)		0.140	2.173	
Male	35 (53.8%)	30 (46.2%)				
Ethnicity						
African	0	8 (100%)		b	b	
Bi-racial (African and Caucasian)	8 (53.3%)	7 (46.7%)				
Caucasian	34 (55.7%)	27 (44.3%)				
Asian^a^	0	1 (100%)				
CBCL 6/18 cluster of symptoms	Univariate analysis			Final multivariate stepwise analysis		
	Odds Ratio	95% Confidence Interval	*p*-value	Odds Ratio	95% Confidence Interval	*p*-value
Challenging and oppositional problems (without X with alteration)						
Sex						
Female/Male	0.56	0.2–1.57	0.267	–	–	–
Ethnicity						
Caucasian/African	1.46	0.33–6.56	0.619	–	–	–
Caucasian/Bi-racial	2.83	0.82–9.76	0.099	–	–	–
Presence of the T allele of the rs6277 SNP of the *DRD2* gene						
No/Yes	2.78	1.15–6.71	0.023	2.78	1.15–6.71	0.023
Attention problems and hyperactivity (few X substantial alterations)						
Sex						
Female/Male	0.66	0.23–1.92	0.444	–	–	–
Ethnicity						
Caucasian/African	0.34	0.04–2.88	0.320	–	–	–
Caucasian/Bi-racial	0.96	0.27–3.37	0.945	–	–	–
Presence of the T allele of the rs6277 SNP of the *DRD2* gene						
No/Yes	2.69	1.001–7.24	0.050	2.69	1.001–7.24	0.050

^a^As there was only one child with Asian ethnicity in the study, this category could not be included in the logistic regression models”; “^b^Degrees of freedom (df) = 3; χ^2^ test could not be performed as there were not the minimum number of observations in each cell

## Discussion

In the present study, the possible associations of the polymorphisms c.957C > T (rs6277) and c.-585A > G (rs1799978) of the *DRD2* gene with the emotional/behavior problem section of the CBCL/6–18 were investigated; a significant association was detected between the polymorphism c.957C > T (rs6277) and the occurrence of oppositional defiant disorders, attention problems, and hyperactivity. The association remained after controlling for sex and ethnicity.

The allele frequency of the SNP c.957C > T (rs6277) was similar to that of the general population [25, 26]. It is interesting to note that the genotype distributions of the African, Asian, and European populations differ from each other as well as from the population of the present study; in particular, in the Asian and African populations, the T/T genotype is extremely rare (6%) and there is a higher frequency of heterozygotes in the European population (54%), indicating heterogeneity among these populations, and similarity between the Brazilian and Amerindian populations [25, 26]. The allelic frequency of the SNP c.-585A > G (rs1799978) in the study population was similar to that of the general population, with a slightly higher MAF of the G allele in the global population (11.9% vs. 11.2%) [26, 27].

Ethnicity did not have statistical influence in the logistic regression models. Moreover, Pena et al. in 2011 showed that the race of individuals in Brazil cannot be predicted from their genomic ancestry alone [28]. Understanding the heterogeneity and admixture of Brazilians within and between geographical regions has important clinical implications for the design and interpretation of clinical trials, practice of clinical genetics and genomic medicine, implementation of pharmacogenetic knowledge in drug prescriptions, and extrapolation of data from other, more homogeneous populations [28]. The admixture proportions vary greatly among Brazilian populations as well as across Latin America [29]. The pooled ancestry contributions in Brazil are reported to be 0.62 European, 0.21 African, and 0.17 Amerindian [29].

The results of the present study showed a higher frequency association amongst externalizing symptoms, which, according to Goodman, result in hyperactive, challenging, aggressive, or antisocial behaviors [30]. These symptoms include conditions of difficulties in impulse control, emotion self-control, and behavior regulation [30, 31]. These conditions share a spectrum of externalization associated with personality dimensions named “disinhibition” and inversely, “retraction” [31–33]. The challenging and oppositional problems as well as the attention and hyperactivity problems are common and potentially harmful [31–33]. They frequently occur as comorbidities and share some common etiological factors. The dopaminergic system influences and regulates diverse neuronal and physiological activities such as the sleep/wake cycle, mechanisms of reward and reinforcement, and motivation and learning, besides modulating voluntary movement control [1, 4, 34, 35].. According to Hirvonen et al. (2009), the C/C genotype is associated with low striatal DRD2 availability (C/C < C/T < TT) [34]. Nevertheless, the authors, in a study of positron emission tomography (PET), found that the C/C genotype was associated with high extrastriatal DRD2 binding potential throughout the cortex and the thalamus (C/C > C/T > T/T) [34]. The authors hypothesized that the putative region-specific features of this SNP could potentially be explained by differential effects of endogenous dopamine on receptor binding, regulation of receptor gene and protein expression or epigenesis, or combination of these factors together with tracer-specific differences [34].

In this study, the presence of the T allele (C/T and T/T) was significantly associated with clinical attention and hyperactivity problems. Deregulation of the reward system has been proposed as a theoretical model contributing to attention deficit hyperactivity disorder, in which failure in the phasic liberation control of dopamine in the striate would result in injury of the cortico-striatal duct, influencing the connection between the cingulate anterior and parietal cortices with the caudate nucleus [36, 37]. According to Dichter et al., dopaminergic function reduction can be implicated with problems in learning and poor behavioral control, but has a reduced influence on rewards related to the behavior [36].

Oppositional defiant disorder involves the violation of others’ rights and of the social rules, and is characterized by recurrent patterns of defiant behavior against authority figures, aggressiveness, and violence; it is frequently associated with attention deficit hyperactivity disorder and other kinds of impulsive behaviors [33, 36, 38, 39]. Dopaminergic polymorphisms have been associated with a variety of negative adaptive and antisocial phenotypes [33, 36]. It has also been suggested that a disability in impulse control is related to a higher tendency toward violence and aggressiveness [33]. The hypo-reactivity of the orbitofrontal cortex and the reduction of dopaminergic function are related to hyposensitivity of the reward system, favoring transgressor behaviors, delinquency, and the abuse of psychoactive substances [39].

A limitation of the present study was the fact that all of the patients were under psychiatric pharmacological treatment during the CBCL assessment. In addition, the parents’ and caregivers’ evaluations regarding the children’s or adolescents’ behaviors tended to focus more on recent problems. The prescription of psychiatric medications as an attempt to decrease externalizing behavioral symptoms can have a positive effect on previously problematic behaviors, and may have influenced the perception in a minority of cases at the time of the CBCL assessment. Nevertheless, the scores of recorded behaviors were high, suggesting that the symptoms were still relevant by the time of the interviews, even with medication use. Another limitation is the small number of participants, which prevents the generalization of the findings. Although preliminary, the findings of this study suggest there may be a potential association of the T allele of the polymorphism c.957C > T SNP with externalized behavioral conditions. Further studies, with a more representative sample, would also include individuals with the G/G genotype of the rs1799978 polymorphism of the *DRD2* gene. This would allow more refined tests for different groups of individuals, including comparisons between those with more than one of the SNPs possibly associated with externalized behavior problems.

The design of this study did not allow to evaluate the effect of the medication on the genotype. The associations found may reflect more treatment response rather than endogenous behavior. Further studies with other designs, including the use of animal models, might aim at exploring the influence of psychopharmacologic treatment on the genotype and on the behavior.

## Conclusions

The association of the T allele of the polymorphism c.957C > T (rs6277) with disruptive/aggressive symptoms, and problems of behavior, oppositional defiance, and attention/hyperactivity suggest that *DRD2* gene expression changes can help with the identification of genetically associated behavioral and mental phenotypes. New studies analyzing the possible longitudinal association between these polymorphisms and the symptoms of more common occurrence in adults (manic psychotic, depressive symptoms) are encouraged to evaluate these relationships in more detail. In particular, more in-depth study of the SNPs are required for applications in not only improving diagnoses but also for preventive medicine. A broader view of the field of externalized behavioral conditions with respect to personalized medicine taking into account different genetic susceptibilities may facilitate the development of new drugs, as well as provide new ways of prescribing existing drugs, in a genetically oriented way according to the needs of each patient.

## Abbreviations

CBCL: Child Behavior Checklist
CBMEG: Molecular Biology and Genetics Engineering Center
DNA: Deoxyribonucleic acid
*DRD2*: Dopamine D2 receptor gene
DSM: Diagnostic and Statistical Manual of Mental Disorders
FAPESP: São Paulo Research Foundation
PET: Positron Emission Tomography
RNA: Ribonucleic acid
SNP: Single Nucleotide Polymorphisms
Unicamp: University of Campinas
